# Editorial

**Published:** 1872-04-15

**Authors:** 


					﻿THE
Medical Examiner
-4 Semi-Monthly Journal of Medical Sciences.
EDITED BY
N. S. DAVIS, M. D., AND F. H. DAVIS, M. D.
Chicago. A/pril 15th, 1S7Q
EDITORIAL.
Medical Books. — We have received a
printed Catalogue, embracing an extensive
list of medical books and monographs, for
sale by James Campbell, 18 Tremont street,
Boston, Mass.
We have also on our table a recent Cata-
logue of valuable medical works published
by the well-known publishing house of Lind-
say & Blakiston, of Philadelphia. Their list
embraces the publications of the Sydenham
Society also.
Cerebro-Spinal Meningitis.—This disease
appears to have been prevailing for some
months past in a large part of the populous
towns through a belt of country embracing
the northern part of Iowa, Illinois, Indiana,
and the southern part of Wisconsin, Michi-
gan, and the western part of New York. In
some places it has been very fatal; in others,
only moderately so.
It continues to prevail, moderately, in this
city; though, we think, less than during the
last part of March and first half of April. In
the present number of the Examiner will be
found a full detail of the cases treated in the
County Hospital in this city; our next issue
will contain an account of the disease as it
appeared in Sterling, Illinois, and its vicinity.
We hope practitioners in other places will
give us the results of their observations con-
cerning the present epidemic.
				

